# Dust storm simulation over Iran using HYSPLIT

**DOI:** 10.1186/2052-336X-12-9

**Published:** 2014-01-07

**Authors:** Khosro Ashrafi, Majid Shafiepour-Motlagh, Alireza Aslemand, Sarmad Ghader

**Affiliations:** 1 Graduate Faculty of Environment, University of Tehran, Tehran, Iran; 2 Institute of Geophysics, University of Tehran, Tehran, Iran

**Keywords:** Dust storms, Dust sources, Trajectory, PM_10_, HYSPLIT, MODIS satellite images

## Abstract

Particulate matters have detrimental effects on human health, environment and economic. This pollutant may emit from anthropogenic or natural sources. On global scale, main proportion of natural particulate matter release to the atmosphere because of wind erosion from arid and semi-arid regions. Recently, the amount of dust coming from Arabian countries has dramatically increased, especially dust storms that are affecting western and even central parts of Iran. This phenomenon has caused a lot of environmental problems. Dust source identification and trajectory simulation using numerical techniques are the main aims of this study. HYSPLIT (Hybrid Single Particle Lagrangian Integrated Trajectory) model dust module and trajectory simulation are utilized in this research and two case studies are investigated (in May and June 2010). The base of the HYSPLIT dust module is the PM_10_ dust storm emission algorithm for desert land use. This methodology is applied to estimate hotspots and trajectories. Due to the results, dust storms started on May 17th and June 7th because of high wind shear (>8.5 m/s) from the western Syrian Desert. The source region limited to 32.50 °N to 33.80 °N and 38.00 °E to 38.80 °E coordinates. Dust plumes lifted and dispersed towards the east and southeast of the sources and reached Ahvaz on May 18th and June 8th. The average of PM10 concentration in these dates reached 625 and 494 $\frac{\mathrm{\mu g}}{m^{3}}$ on Ahvaz monitoring stations, respectively. Moreover, the results gained from the model for dust motion simulation are similar to the MODIS satellite images.

## Introduction

**Figure 1:**
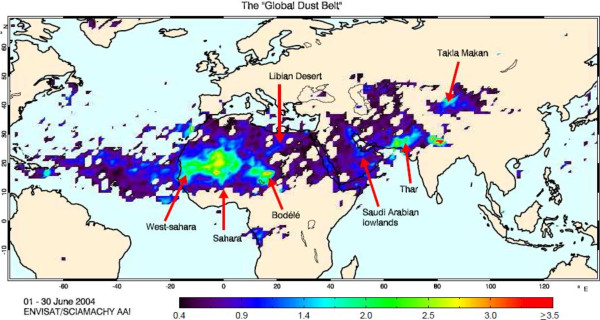
**Dust belt in SCIAMACHY**^**a **^**absorbing aerosol index **[5]**.** (Figure courtesy of M. de Graaf).

Dust storms and wildfires in natural resources can be the considerable sources of suspension dust [1]. On global scale, most of the dust emission comes from arid and semiarid areas [2]. The main dust source areas are located in arid climates (with annual rainfall under 200–250 mm) in the so-called ‘dust belt’ [3]. The ‘dust belt’ extends from the west coast of North Africa, the Middle East, central and south Asia to China (Figure 1) [4].

There are many drawbacks with the dust phenomenon such as environmental, socio-economic, human health, climate and microclimate problems [6]. Some of these issues are discussed as follows.

Wind-blown dust is an effective factor for the transport of pathogens and pollutants [7,8] and also can influence air quality downwind of dust source regions by reducing visibility, soiling property and causing illnesses [9,10]. Inhalation of dust particles can cause heart beat irregularities, heart attacks and respiratory problems, severe and chronic headaches, severe allergies and skin diseases [11].

Particles such as mineral dust, by absorbing ultraviolet radiation can inhibit smog production, having profound implications in the control of air pollution in urban areas [12]. Furthermore, the interactions between wind-blown dust and anthropogenic pollutants aggravate the generation of secondary aerosols [13].

Dust particles have a significant effect on climate, acting both directly (by scattering and absorbing radiation) and indirectly (by changing the optical properties of clouds) on the Earth’s radiation balance [14]. Absorption and scattering of solar radiation caused by dust events may affect air temperatures [15]. In another way Dust fertilization (including iron and phosphorus) of poor nutrient marine environments can increase formation of phytoplankton and can influence the global cycle of carbon [16].

Different Techniques have been developed to identify dust hotspots and pathways. Numerical modeling, trajectory analysis, Remote sensing and satellite imagery, dust observations and metrological data analysis, mineral tracers and geological models can be applied as the principal tools used to research dust events [4,6,17-20].

In Iran a few studies have been carried out to determine dust sources, trajectories, contribution of dust to urban PM_10_ concentrations and temporal and spatial coverage of dust by using modeling techniques [21]. It should be mentioned that most of the conducted studies on these issues used satellite images and meteorological data analysis [22,23]. Most of the Dust storms in Iran are coming from the western and southern neighboring countries and they affect western and central regions of Iran [6].

In this research, the most high risk city of Iran namely Ahvaz, is chosen as the case study. According to WHO database [24], Ahvaz with 372 $\frac{\mathrm{\mu g}}{m^{3}}$ annual mean of concentration of particulate matter is the first polluted city in the world. Finding the dust sources and the trajectories which cause the dust in Iran (specially the cities of Ahvaz and Tehran) has a significant importance. Therefore, this research uses numerical modeling techniques to study meteorological parameters, sources and trajectories of suspended particles of dust storms from wind erosions events. Surly, the results aim to control and reduce the amount of pollutions.

## Materials and methods

### Study area

**Figure 2:**
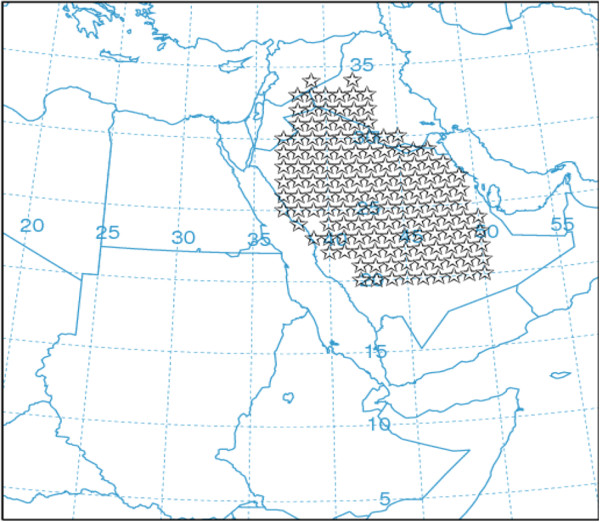
Dust sources regions defined in HYSPLIT dust module.

Study area for dust injection to the atmosphere investigated using HYSPLIT^b^ model including parts of Saudi Arabia, Iraq, Syria and Jordan. This area limited to 20 °N −36.30 °N and 37 °E - 50 °E coordinates. Only desert land-use areas participate in the dust module and model omits the other land uses automatically by definition of this algorithm. Figure 2 shows the desert land-use considered for dust emission in the present study.

### HYSPLIT model description

The HYSPLIT model uses puff or particle approaches to compute trajectories, complex dispersion and deposition. The model computation method is a combination of Eulerian (concentrations are calculated for each grid cell using integration of pollutant fluxes at every grid cell interface due to advection and diffusion) and Lagrangian (concentrations are computed by summing the contribution of each pollutant “puff” that is advected through the grid cell as represented by its trajectory) approaches.

The model utilizes meshed meteorological data on one of three conformal map projections (Polar, Lambert and Mercator). The dispersion model requires meteorological data fields that can be obtained from archives or from forecast model outputs and the datasets should be formatted for input to HYSPLIT [25,26].

The accuracy of the model is considerably dependent on the meteorological data resolution [27], For this study, we used GDAS^c^ meteorological data provided by U.S NOAA.

In HYSPLIT dust module, PM_10_ dust injections are estimated using as mass source algorithm [28].

(1) $$F=K\frac{\rho }{g}u_{*}\left(u_{*}{}^{2}-u^{2}{}_{*t}\right)$$

where F is the vertical mass flux of dust that is obtained from the friction velocity u_*_, a threshold friction velocity u_*t_, (required for initiation of dust emission), and a coefficient *K* (s/m) that depends on the surface soil texture. The friction velocity varies in space and time. However, the threshold velocity and soil texture coefficient vary only in space and related to the soil, land-use characteristics and surface roughness. In this study, the model is used over domain where detailed soil characteristics are not available and revised version of the dust module for vertical mass flux is replaced as Equation (1) [29].

(2) $$F=0.01u_{*}{}^{4}$$

Based on these algorithms PM_10_ is emitted from desert land-use, when the wind velocity exceeds from local friction velocity and this parameter is defined as:

(3) Ut=u*tklnzz0ns

Where *z*_*0ns*_ is the aerodynamic roughness length for non-saltating conditions, z is the wind measurement height and Von Karman’s constant *k* is assumed to be 0.4 [17].

In this research, dispersion simulation is done over the study area with HYSPLIT dust module. A horizontal domain of 30° × 30° with resolution of 0.05^◦^ × 0.05^◦^ and a vertical level of 100 meters above ground level is considered in dispersion model. Pollutant concentrations are sampled in every time step and are averaged over every 12 h. The turbulence mixing is computed using a diffusivity approach based on vertical stability estimates and the horizontal wind field deformation. The puff dispersion is assumed to be linear function of time. Ground level concentrations are calculated as average of the lowest 100 m within each horizontal grid cell. HYSPLIT dust storm modeling done for 0.25° × 0.25° resolution for desert dust sources and a total of 100,000 particles or puffs are released during one release cycle with a maximum of 50,000 particles permitted to be carried at any time during the simulation. Release mode is sampled with 3-dimensional (3-D) particle horizontal and vertical option.

The trajectory calculation in any Lagrangian model is based on the following the particle or puff. Therefore, once the basic meteorological data (U, V and W) has been processed and interpolated to the model grid. Trajectories can be computed to test the advection components of the model. The advection is computed from the average of the 3-D velocity vectors for the initial-position P(t) and the first-guess position P’(t + Δt). The velocity vectors are linearly interpolated in both space and time [25,26].

The first guess position is

(4) $$P'\left(t+\Delta t\right)\;=\;P\left(t\right)+V\left(P,t\right)\;\Delta t$$

and the final position is

(5) $$P\left(t+\Delta t\right)=P\left(t\right)+0.5\;\left[\;V\left(P,t\right)+V\left(P',t+\Delta t\right)\;\right]\;\Delta t$$

In this study back trajectory simulations were used for determining source of dust storms and motion direction of dust plume over Middle East and Iran. Back trajectories started from Ahvaz (31.24 °N, 48.49 °E) and Tehran (35.42 °N, 51.25 °E) at the time of dust arrival. For HYSPLIT trajectory setting, four trajectory tracking levels including 500, 1000, 2000 and 3000 m are considered and also the top of model assumed to be 10,000 m.

Turbulence, wind fields and mixing depth values are used as inputs for dispersion model.

## Results and discussion

First, the meteorological parameters surveyed in desert areas and the results showed that high wind velocity and mixing height caused to inject and lift the dust to the atmosphere, respectively. Second, dust hotspots determined in Syrian deserts and motion of dust simulated over study area using meteorological fields. The results are discussed in details as follows.

Two case studies are simulated in this research. These dust storms occurred on May 17^th^ -20^th^ and June 7^th^ - 10^th^, 2010. In these two periods of time, PM_10_ concentration dramatically exceeded the ambient air quality standards (50 $\frac{\mathrm{\mu g}}{m^{3}}$). Table 1 Shows PM_10_ concentrations in three air quality stations of Ahvaz during these periods [30].

**Table 1:** **PM**_**10 **_**concentrations in Ahvaz air quality stations (**$\frac{\mathrm{\mu g}}{m^{3}}$**)**

**Date of dust event**	**Environment dept. station**	**Meteorology office station**	**Naderi station**
17-May	128	88	167
18-May	497	483	508
19-May	339	383	383
20-May	438	508	508
7-Jun	275	817	100
8-Jun	683	1100	100
9-Jun	215	204	33
10-Jun	266	254	158

### Meteorogical fields

**Figure 3:**
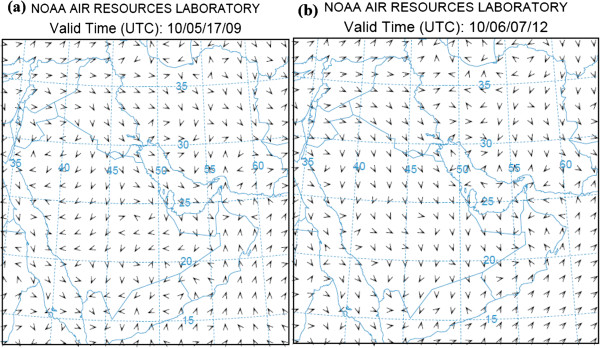
Wind fields over study area at the time of dust injection starting on (a) May 17th and (b) June 7th.

Meteorological fields and flow patterns are analyzed in the 3-D model grid. Meteorological simulation indicates the development of diurnally varying local flow pattern. The wind fields at 10 m above the ground surface at 9 UTC on May 17^th^ (a) and 12 UTC on June 7^th^ 2010 (b) are shown in Figure 3. As illustrated in the figure, the main flow pattern was westerly and northwesterly in dust region around midday. This pattern rarely changed in the course of the day.

Table 2 shows the case studies’ meteorological parameters in dust hotspots area at the time of injection. As indicated in Table 2, these parameters are approximately similar in two assessed dust events. The wind speed is the most important parameter that influences particulate matter advection and dispersion and planetary boundary layer height (PBLH) Causes particulates dispersing in vertical direction. In these events PBLH was above 2000 m and dust dispersed effectively in vertical direction.

**Table 2:** Surface metrological parameters for two dust events in source areas

**Parameter**	**Pressure (hpa)**	**Surface height (m)**	**Temperature at 2 m (c)**	**U wind**^**d **^**at 10 m (m/s)**	**V wind**^**e **^**at 10 m (m/s)**	**PBLH (m)**
May event	936	676	34.4	8.6	0.75	2063
June event	925	740	31.7	10.1	2.2	2576

### Dust module modeling results

May dust event was started at about 9 UTC on May 17th from west of syrian desert and dust injected for about 15 hours. Main hotspots derived by model for case studies are shown in Table 3. Dust plume spreaded in northwesterly direction and reached to Ahvaz in noontime of May 18 and increased PM_10_ concentration in the city to 496 $\frac{\mathrm{\mu g}}{m^{3}}$ reported by DOE. Dust storm reached to Tehran on May 19th at 6 UTC and air quality of Tehran drastically was affected by air borne particles. The avarege of PM_10_ concentration of Tehran was 343 $\frac{\mathrm{\mu g}}{m^{3}}$.

**Table 3:** Dust hotspots resulted by HYSPLIT dust module

**May event hotspots**	**(32.50 °N, 38.00 °E)**	**(33.80 °N, 38.30 °E)**	**(32.50 °N, 38.25 °E)**
June event hotspots	(33.75 °N, 38.25 °E)	(33.75 °N, 38.80 °E)	-

**Figure 4:**
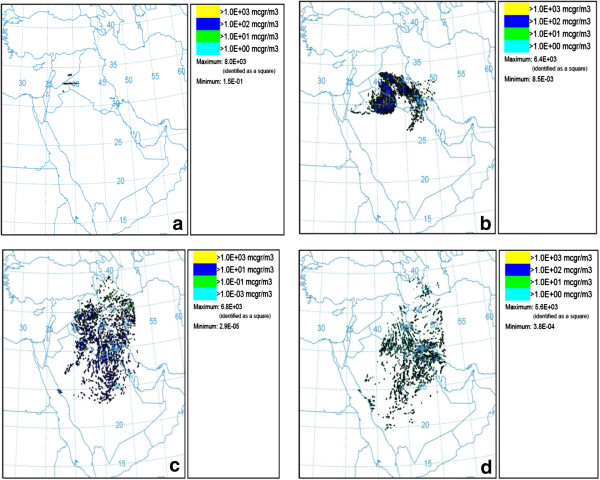
Modeling results for concentration PM10 of averaged 0 – 100 m in May, (a) 17, (b) 18, (c) 19 and (d) 20 May.

**Figure 5:**
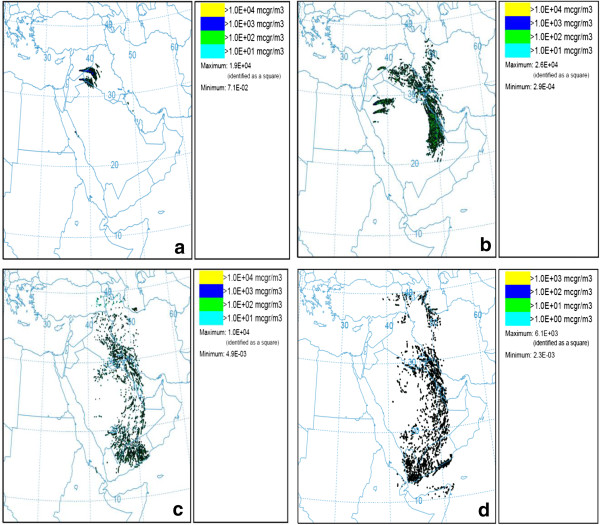
Modeling results for concentration of PM10 averaged 0 – 100 m in June, (a) 7, (b) 8, (c) 9 and (d) 10 June.

In the second case study, when wind speed exceeded 10.34 m/s in Syrian Desert (Syria and Jordan region) at 12 UTC on June 7th, dust storm started and dust emitted for about 12 hours. This event affected western part of Iran, especially Khuzestan province. Dust plumes reached to Ahvaz at the beginning of the June 8th. The average of PM_10_ concentration reached to 625 $\frac{\mathrm{\mu g}}{m^{3}}$ in the Ahvaz monitoring stations during the day. Figures 4 and 5 show results of dust storm modeling for May (17 May (a), 18 May (b), 19 May (c) and 20 May (d)) and June (7 June (a), 8 June (b), 9 June (c) and 10 June (d)) dust events, respectively.

### Trajectory results

**Figure 6:**
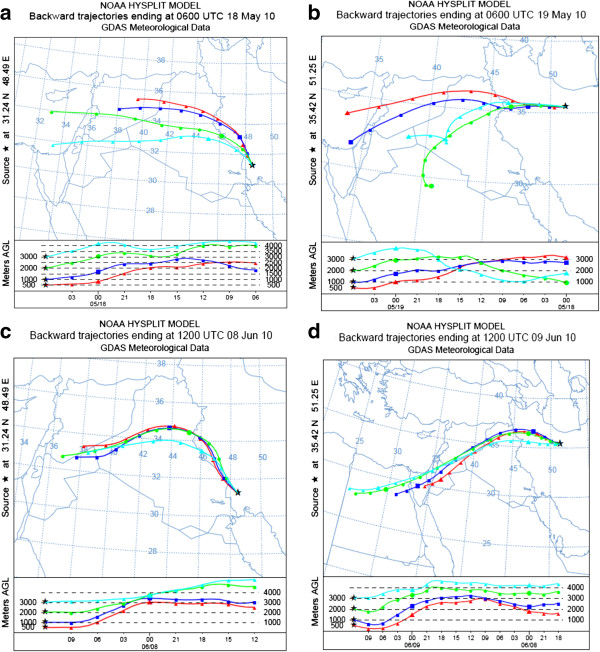
**HYSPLIT Back trajectory simulations. (a)** Ahvaz on May 18th, **(b)** Tehran on May 19th, **(c)** Ahvaz on June 8th, **(d)** Tehran on June 9th.

In this research, Horizontal and vertical pathways of dust parcel are simulated using trajectory approach. For May dust event, according to the arrival time of dust storms to Ahvaz and Tehran, back trajectory simulation started at 6 UTC on May 18th for Ahvaz and 6 UTC on May 19th for Tehran. The source of dust storm was located in west and north of Syrian Desert. Trajectory simulation began at 12 UTC on June 8th for Ahvaz and 12 UTC on June 9th for Tehran for June case study. This trajectory modeling showed that Source of early June dust storm was in western Syrian Desert and near the border of Syria, Jordan and Saudi Arabia. Figure 6 shows backward trajectory plots of Tehran and Ahvaz (Ahvaz at 6 UTC 18 May (a), Tehran at 6 UTC 19 May (b), Ahvaz at 12 UTC 08 June (c) and Tehran at 12 UTC 09 June (d)).

In the first case in May, studying arrival altitudes in Tehran and Ahvaz indicates that vertical distribution of dust in Tehran was 1000 and in Ahvaz was about 2000–3000 m. in the second case in June; height of dust plume was up to 2000 m above ground surface.

**Figure 7:**
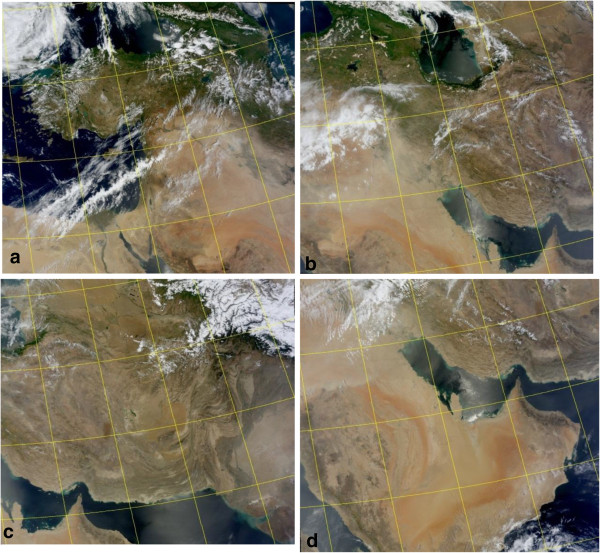
MODIS satellite images for May dust storm. (a) 17, (b) 18, (c) 19 and (d) 20 May.

**Figure 8:**
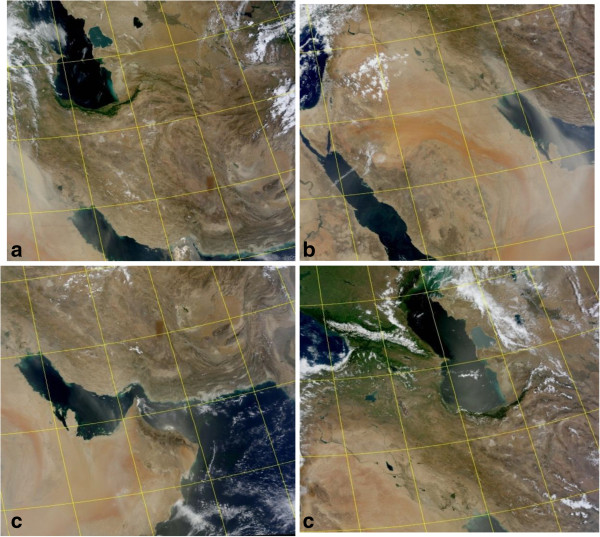
MODIS satellite images for June dust storm. (a) 7, (b) 8, (c) 9 and (d) 10 June.

For supporting HYSPLIT results, MODIS^f^ satellite images have been used in this study. MODIS plays a significant role on earth observations of dust events which can identify dust storms using optical satellite images based on the radiation and scattering characteristics of particles. Temporal and spatial coverage computed by HYSPLIT was significantly similar to MODIS images. Figures 7 and 8 demonstrate MODIS images for case studies over study area provided by NASA rapid response imagery [31].

According to Figure 7, on May 17^th^, dust appeared in Syria and after passing over Iraq gradually moved to the western and central parts of Iran. Also Figure 8 shows that dust storm on June 7th after covering large regions of Iraq reached Khuzestan province and finally circulated towards the Persian Gulf and Arabian Peninsula.

In this study, MODIS images confirmed the results of HYSPLIT dust modeling and it was detected that dust plumes had a circulating motion while moving towards eastern parts of Middle East.

## Conclusion

In this research source identification and trajectory simulation of two dust storms over Iran which was caused by wind erosion are studied. The HYSPLIT model dust module was applied to a western Middle East deserts. However, the results clarified that both of dust events simulated started from Syrian Desert in similar coordinates. Due to the high shear wind speed and mixing height, dust was released from desert land-use and dispersed horizontally and vertically over the study area. In addition, strong winds transported the dust through large areas of Iraq and Kuwait reaching the significant parts of Iran in about 48 hours. Backward trajectory simulation from Tehran and Ahvaz confirmed dust sources derived by dust module. At end, dust motion in MODIS images were compared to the output of HYSPLIT simulation which showed same trends.

## Endnotes

^a^Scanning Imaging Absorption Spectro-Meter for Atmospheric Cartography

^b^Hybrid Single-Particle Lagrangian Integrated Trajectory

^c^Global Data Assimilation System

^d^Zonal component of wind

^e^Meridional component of wind

^f^Moderate Resolution Imaging Spectro radiometer

## Competing interests

The authors declare that no conflict of interest.

## Authors’ contributions

All authors read and approved the final manuscript.

## References

[B1] Wen Kuoa H Yi Shena H Indoor and outdoor PM2.5 and PM10 concentrations in the air during a dust storm Build Environ 2010 3 45 610 614

[B2] Shao Y Physics and Modeling of Wind Erosion 2008 Germany: Springer Press

[B3] Prospero M Ginoux P Torres O Nicholson E Gill E Environmental characterization of global source of atmospheric soil dust identified with the nimbus 7 total ozone mapping Spectrometer (TOMS) absorbing aerosol product J Geophys Res 2002 40 1 1 31

[B4] Escudero M Stein A Draxler R Querol X Alastuey A Castillo S Determination of the contribution of north Africa dust source areas to PM10 concentrations over the central Iberian peninsula using the hybrid single-particle lagrangian integrated trajectory model (HYSPLIT) model J Geophys Res 2006 111 D06210 DOI: 10.1029/2005JD006395

[B5] de Graaf M Remote Sensing of UV-absorbing aerosols using space-borne spectrometers, Ph.D Thesis 2006 Amsterdam: Vrije Universiteit Amsterdam 132

[B6] Gerivani H Lashkaripour G Ghafoori M Jalali N The source of dust storm in Iran: a case study based on geological information and rainfall data Carpathian J Earth Environ Sci 2003 6 1 297 308

[B7] Shinn EA Smith GW Prospero JM Betzer P Hayes ML Garrison V Barber RT African dust and the demise of Caribbean coral reefs Geophys Res Lett 2001 27 19 3029 3032

[B8] Wang Y Zhuang G Tang A Zhan W Sun Y Wang Z An Z The evolution of chemical components of aerosols at five monitoring site of China during dust storms Atmos Environ 2007 41 1091 1106 10.1016/j.atmosenv.2006.09.015

[B9] McKendry G Hacker P Stull R Sakiyama S Mignacca D Reid K Long-range transport of Asian dust to the lower Fraser valley, British Columbia, Canada J Geophys Res 2001 106 18,361 18,370 10.1029/2000JD900359

[B10] Chan Y McTainsh G Leys J McGowan H Tews K Influence of the 23 October 2002 dust storm on the air quality of four Australian cities Water Air Soil Pollut 2005 164 329 348 10.1007/s11270-005-4009-0

[B11] Griffin D Kellogg C Dust storms and their impact on ocean and human health: dust in Earth’s atmosphere Ecohealth 2004 1 284 295

[B12] Dickerson R Kondragunta S Stenchikov G Civerolo K Doddridge B Holben B The impact of aerosols on solar ultraviolet radiation and photochemical smog Science 1997 278 827 830 10.1126/science.278.5339.827 9346474

[B13] Alastuey A Querol X Castillo S Escudero M Avila A Cuevas E Torres C Romero PM Exposito F Garcia O Diaz JP Dingenen RV Putaud JP Characterization of TSP and PM2.5 At izanea and Sta. Cruz de Tenerife (canary islands, Spain) during a Saharan dust episode (July 2002) Atmos Environ 2005 39 26 4715 4728 10.1016/j.atmosenv.2005.04.018

[B14] IPCC Fourth Assessment Report of the Intergovernmental Panel on Climate Change. Working Group I: the Physical Science Basis of Climate Change. Summary for Policymakers 2007 Geneva, Switzerland: Intergovernmental Panel on Climate Change

[B15] Miller RL Tegen I Climate response to soil dust aerosols J Clim 1998 11 3247 3267 10.1175/1520-0442(1998)011<3247:CRTSDA>2.0.CO;2

[B16] Gao Y Fan S Sariento JL Aeolian iron input to the ocean through precipitation scavenging: a modelling perspective and its implications for natural iron fertilization in the ocean J Geophys Res 2003 108 7 4221

[B17] Draxler R Gillette A Kirkpatrick S Heller J Estimating PM10 air concentrations from dust storms in Iraq, Kuwait and Saudi Arabia Atmos Environ 2001 35 4315 4330 10.1016/S1352-2310(01)00159-5

[B18] Xuan J Emission inventory of eight elements, Fe, Al, K, Mg, Mn, Na, Ca and Ti, in dust source region of East Asia Atmos Environ 2005 39 813 821 10.1016/j.atmosenv.2004.10.029

[B19] Wang W Fang ZY Numerical simulation and synoptic analysis of dust emission and transport in East Asia Glob Planet Chang 2006 52 57 70 10.1016/j.gloplacha.2006.02.004

[B20] Alam K Qureshi S Blaschke T Monitoring Spatio-temporal aerosol patterns over Pakistan based on MODIS, TOMS and MISR satellite data and a HYSPLIT model Atmos Environ 2011 45 4641 4651 10.1016/j.atmosenv.2011.05.055

[B21] Givehchi R Arhami M Tajrishy M Contribution of the middle eastern dust source areas to PM10 levels in urban receptors: case study of Tehran, Iran Atmos Environ 2013 75 287 295

[B22] Zolfaghari H Abedzadeh H Synoptic analysis of dust sources in west of Iran Journal of Geography and Development 2005 3 6 173 188 (in Persian)

[B23] Iranmanesh F Akram M Survey on sources areas and characteristics of dust storms dispersion in sistan region using satellite imagery processing Construction and research journal 2003 67 104 (in Persian)

[B24] WHO Urban Outdoor air Pollution Database. Department of Public Health and Environment 2011 Geneva, Switzerland: World Health Organization http://www.who.int/phe/health_topics/outdoorair/databases/OAP_database.xls

[B25] Draxler R Hess GD An overview of the HYSPLIT_4 modeling system for trajectories, dispersion and deposition Aust Meteorol Mag 1998 47 295 308

[B26] Draxler R Stunder B Rolph G Stein A Taylor A Hybrid Single-Particle Lagrangian Integrated 2009 United States: NOAA

[B27] Challa VS Indrcanti J Baham JM Patrick C Rabarison MK Young JH Hughes R Swanier SJ Hardy MG Sensitivity of atmospheric dispersion simulations by HYSPLIT to the meteorological predictions from a meso-scale model Environ Fluid Mech 2008 8 4 387 367 trajectories 4 user’s guide. NOAA Tech. Memo, ERL-ARL

[B28] Marticorena B Bergametti G Gillette D Belnap J Factors controlling threshold friction velocity in semiarid and arid areas of the United States J Geophys Res 1997 102 23,277 23,287 10.1029/97JD0130311541236

[B29] Westphal L Toon OB Carlson TN A two-dimensional numerical investigation of the dynamics and microphysics of Saharan dust storms J Geophys Res 1987 92 3 3027 3049

[B30] IR.DOE data Ahvaz air Pollution Monitoring Stations Datasets 2011 Tehran, Iran: Department of Environment

[B31] MODIS rapid response Satellite Images 2010 http://rapidfire.sci.gsfc.nasa.gov/realtime/2010158/

